# Rehabilitation and release of orphaned Eurasian lynx (*Lynx lynx*) in Europe: Implications for management and conservation

**DOI:** 10.1371/journal.pone.0297789

**Published:** 2024-03-07

**Authors:** Anja Molinari-Jobin, Fridolin Zimmermann, Stéphanie Borel, Luc Le Grand, Elena Iannino, Ole Anders, Elisa Belotti, Ludek Bufka, Duško Ćirović, Nolwenn Drouet-Hoguet, Thomas Engleder, Michał Figura, Christian Fuxjäger, Eva Gregorova, Marco Heurich, Sylvia Idelberger, Jakub Kubala, Josip Kusak, Dime Melovski, Tomma Lilli Middelhoff, Tereza Mináriková, Paolo Molinari, Lorane Mouzon-Moyne, Gilles Moyne, Robert W. Mysłajek, Sabina Nowak, Janis Ozolins, Andreas Ryser, Bardh Sanaja, Maryna Shkvyria, Teodora Sin, Magda Sindičić, Vedran Slijepčević, Christian Stauffer, Branislav Tám, Aleksander Trajce, Josefa Volfová, Sybille Wölfl, Diana Zlatanova, Kristina Vogt

**Affiliations:** 1 Stiftung KORA, Ittigen, Switzerland; 2 Department of Ecology and Evolution, University of Lausanne, Lausanne, Switzerland; 3 Vetsuisse Faculty, Departement of Infectious diseases and Pathobiology, Institute for Fish and Wildlife Health, University of Bern, Bern, Switzerland; 4 Progetto Lince Italia, Tarvisio, Italy; 5 Harz National Park, Wernigerode, Germany; 6 Faculty of Forestry and Wood Sciences, Czech University of Life Sciences Prague, Prague, Czech Republic; 7 Department of Nature Protection, Šumava National Park Administration, Kašperské Hory, Czech Republic; 8 Faculty of Biology, University of Belgrade, Belgrade, Serbia; 9 Equipe Loup-Lynx, Office français de la biodiversité, Gières, France; 10 Green Heart of Europe, Lynx Project Austria Northwest, Haslach an der Mühl, Austria; 11 Association for Nature "Wolf", Twardorzeczka, Poland; 12 Faculty of Biology, Department of Ecology, Institute of Functional Biology and Ecology, University of Warsaw, Biological and Chemical Research Centre, Warszawa, Poland; 13 Nationalpark Kalkalpen, Molln, Austria; 14 Bojnice Zoological Garden, Bojnice, Slovakia; 15 Institute for Forest and Wildlife Management, Inland Norway University of Applied Sciences, Koppang, Norway; 16 Wildlife Ecology and Wildlife Management, University of Freiburg, Freiburg, Germany; 17 Stiftung Natur und Umwelt Rheinland-Pfalz, Mainz, Germany; 18 Faculty of Forestry, Department of Applied Zoology and Wildlife Management, Technical University in Zvolen, Zvolen, Slovakia; 19 DIANA–Carpathian Wildlife Research, Banská Bystrica, Slovakia; 20 Faculty of Veterinary Medicine, Department Veterinary Biology, University of Zagreb, Zagreb, Croatia; 21 Macedonian Ecological Society, Skopje, North Macedonia; 22 ALKA Wildlife, Dačice, Czech Republic; 23 Faculty of Environmental Sciences, Czech University of Life Sciences Prague, Prague, Czech Republic; 24 Centre Athenas Wildlife Rescue Center, L’étoile, France; 25 Latvian State Forest Research Institute ‘‘Silava”, Salaspils, Latvia; 26 Environmentally Responsible Action ERA, Peja, Kosovo; 27 Kyiv zoological park of national importance, Kyiv, Ukraine; 28 Association for the Conservation of Biological Diversity, Focsani, Romania; 29 Faculty of Veterinary Medicine, Department for Game and Wildlife, University of Zagreb, Zagreb, Croatia; 30 Department of Wildlife Management and Nature Protection, Karlovac University of Applied Sciences, Karlovac, Croatia; 31 Faculty of Agrobiology and Food Resources, Department of Small Animal Science, Slovak University of Agriculture, Nitra, Slovakia; 32 Protection and Preservation of Natural Environment in Albania, Tirana, Albania; 33 Friends of the Earth Czech Republic—Carnivore Conservation Programme, Olomouc, Czech Republic; 34 WildLink Institute, Association Lynx Bavaria, Waldmünchen, Germany; 35 Faculty of Biology, Department of Zoology and Anthropology, Sofia University "St. Kliment Ohridski", Sofia, Bulgaria; Cheetah Conservation Fund, Namibia University of Science and Technology, NAMIBIA

## Abstract

Rehabilitation of injured or immature individuals has become an increasingly used conservation and management tool. However, scientific evaluation of rehabilitations is rare, raising concern about post-release welfare as well as the cost-effectiveness of spending scarce financial resources. Over the past 20 years, events of juvenile Eurasian lynx presumably orphaned have been observed in many European lynx populations. To guide the management of orphaned lynx, we documented survival, rehabilitation and fate after the release and evaluated the potential relevance of lynx orphan rehabilitation for population management and conservation implications. Data on 320 orphaned lynx was collected from 1975 to 2022 from 13 countries and nine populations. The majority of orphaned lynx (55%) were taken to rehabilitation centres or other enclosures. A total of 66 orphans were released back to nature. The portion of rehabilitated lynx who survived at least one year after release was 0.66. Release location was the best predictor for their survival. Of the 66 released lynx, ten have reproduced at least once (8 females and 2 males). Conservation implications of rehabilitation programmes include managing genetic diversity in small, isolated populations and reintroducing species to historical habitats. The lynx is a perfect model species as most reintroduced populations in Central Europe show significantly lower observed heterozygosity than most of the autochthonous populations, indicating that reintroduction bottlenecks, isolation and post-release management have long-term consequences on the genetic composition of populations. The release of translocated orphans could be a valuable contribution to Eurasian lynx conservation in Europe. It is recommended to release orphans at the distribution edge or in the frame of reintroduction projects instead of a release in the core area of a population where it is not necessary from a demographic and genetic point of view. Rehabilitation programmes can have conservation implications that extend far beyond individual welfare benefits.

## Introduction

The International Wildlife Rehabilitation Council defines wildlife rehabilitation as “the treatment and temporary care of injured, diseased, and displaced indigenous animals, and the subsequent release of healthy animals to appropriate habitats in the wild” [1]. The goal of wildlife rehabilitation is to provide professional care and a safe environment to wild animals so that, ultimately, they can be returned to their natural habitat. The most common aims of animal rehabilitation are animal welfare and conservation [2]. Rehabilitation of injured, debilitated or immature individuals has become an increasingly used conservation and management tool [3,4]. As an example for the order of magnitude of wildlife rehabilitation, Grogan and Kelly [5] estimated that in 2011 in England and Wales, more than 70.000 animals (more than 80% of which were birds) were admitted to wildlife rehabilitation centers, with 40% being re-released. However, scientific evaluation of such rehabilitations is rare, raising concern about post-release welfare as well as cost-effectiveness of spending scarce financial resources [6]. Rehabilitation needs to be evaluated with respect to its use for species conservation and carefully implemented with reference to behavioural development and human habituation before release, as both factors can affect behavioural responses later in life [7]. This is especially important when dealing with large carnivores, as they can potentially pose danger to property or harm people. Other challenges of rehabilitation are escaping from enclosures, anesthetic or treatment complications, stereotypic behaviour, self-injuries and stress [8,9].

Only few studies manifest a conservation benefit of large carnivore rehabilitation [9–11]. Rehabilitation of carnivores is controversial due to high costs, supposed large post-release movements, reduced survivorship, and possible conflict at the release site [8], often resulting in management agencies being reluctant in engaging in large carnivore rehabilitation. However, with social media instantaneously spreading information about the appearances of animals in distress, the demand for effective and professional responses will only grow in the foreseeable future. This “instantaneous spotlighting” represents both a problem and an opportunity. The public will expect responses, and the absence of an appropriate response will often be deemed unacceptable [12].

**Fig 1:**
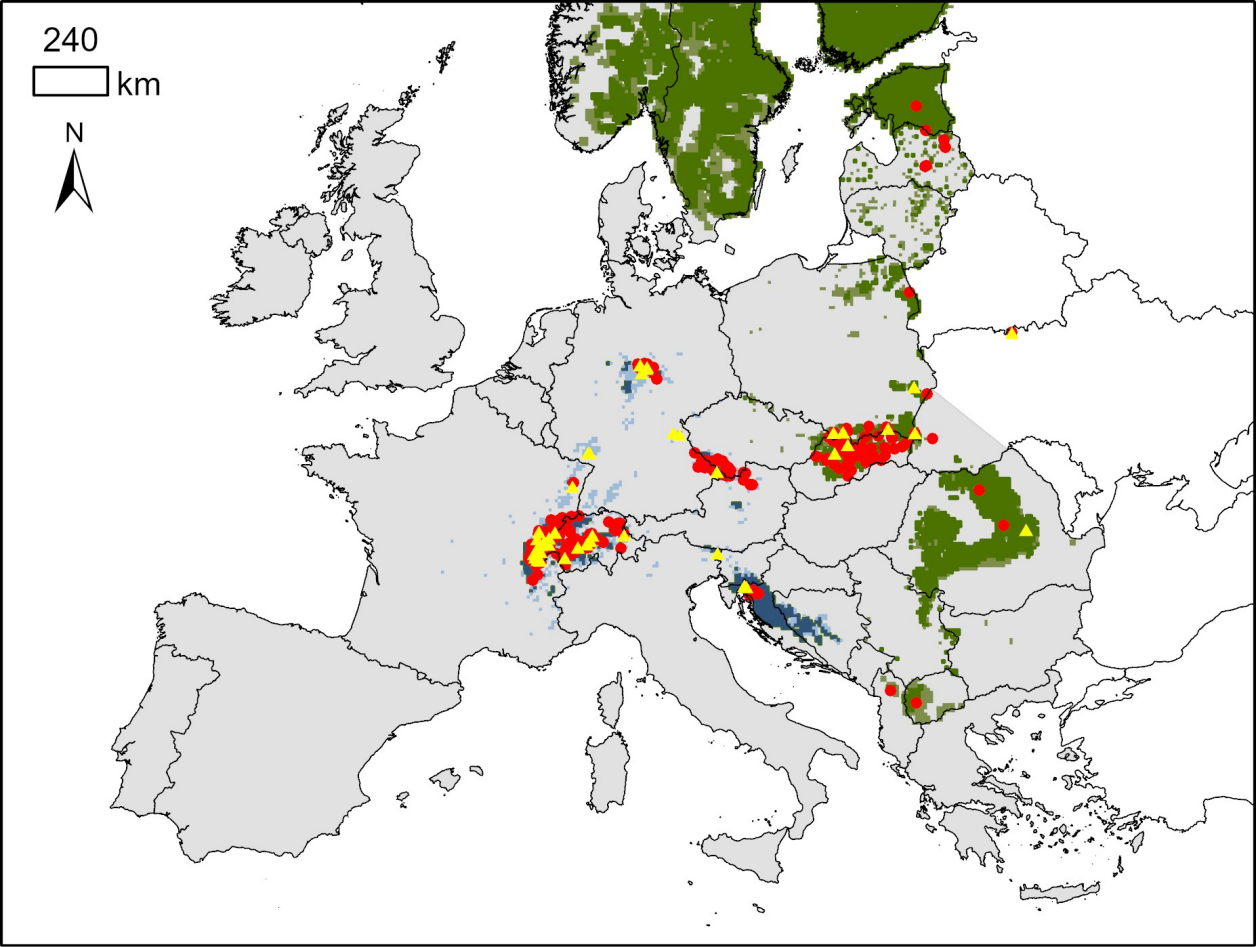
Distribution of reintroduced (blue) and autochthonous (green) lynx populations in Europe based on the survey from 2012–2016 [16]. Darker colours correspond to permanent presence and lighter colours to sporadic presence. Surveyed countries and regions are shown in grey. Red dots indicate occurrence of orphaned lynx and yellow triangles release sites of rehabilitated orphans.

The Eurasian lynx (*Lynx lynx*) had experienced a population minimum in Europe in the middle of the 20^th^ century when its distribution range was reduced to five populations, i.e., Scandinavian and Karelian in the north, Baltic and Carpathian in the east and Balkan in the south [13]. Since the 1970s, the return of the lynx to Central Europe was initiated based on reintroduction projects that resulted in six additional lynx populations (Fig 1). Population sizes in 2016 ranged from a few individuals in the Vosges-Palatinian population to 2.100–2.400 individuals in the Carpathian population (https://www.lcie.org/Large-carnivores/Eurasian-lynx). Especially in small populations, even a few additional individuals can be crucial in achieving population recovery and the survival of each individual is essential, particularly in the early phases of reintroduction programmes [14]. Therefore, in addition to animal welfare aspects, orphan rescue and rehabilitation can have important conservation implications and serve as an important tool for the conservation of small, reintroduced populations [15].

The lynx follows a monoestrus reproductive cycle where ovulation generally occurs once a year [17]. The mating season is mid-February to early April and kittens are usually born in May and June [18]. For the first 6–8 weeks after giving birth, females show a foraging behaviour centred around a den [19,20]. In August, kittens begin to accompany their mother and start to consume meat [21]. Juvenile lynx usually separate from their mother at the age of 10–11 months in March/April [22]. Juvenile animals may become orphans for various reasons: they may lose their mother (trauma, illegal killing, disease), they may fail to follow her due to injury or illness, and they may be abandoned due to suboptimal health conditions or a lack of experience of their mothers. Furthermore, young lynx may be temporarily separated from their family group and assumed to be orphaned [23]. As in nature it is most often impossible to know with certainty whether the mother has died, we hereafter refer to all these cases together as “orphans”. Over the past 20 years, events of juvenile lynx presumably orphaned have been observed in many European lynx populations. Typically, young lynx were found alone in proximity to human settlements, in poor body condition, looking for easily accessible food sources and thus thought to be orphaned [23].

The management of lynx orphans remains a challenge. Up to now, management has often been based on ad-hoc decisions. Four possibilities theoretically exist: rehabilitation, permanent captivity (which may include captive breeding), euthanasia, or care provided in the wild [23]. One or more wildlife rehabilitation centres suitable for lynx are currently available in five of 25 European countries with lynx presence (France, Germany, Poland, Romania, Slovakia). Lynx rehabilitation is presently mentioned only in three management documents [24–26], indicating a lack of guidance on the topic. Only a few studies have evaluated the success of measures taken [23], and existing management plans provide little guidance on best practices. The following assessment aimed to provide data on orphaned lynx handling in Europe, to document survival, rehabilitation and fate after the release and evaluate the potential relevance of lynx orphan rehabilitation for population management and conservation implications. Specifically, we address the following questions: 1) Where do lynx orphans occur and what are the circumstances of appearance of lynx orphans; 2) which management practice increases the survival probability of lynx orphans and subsequently contributes most to species conservation; and 3) what were the dispersal distances of released orphans, how high was their survival, what were the causes of mortality and how many of them have reproduced.

## Methods

Data on individual orphans were collected through a questionnaire which included questions on (1) traits of the individual and circumstances of its discovery; (2) the fate of the specific individual (death, euthanasia, placement in a zoo, escape, release in nature or in-situ feeding); (3) the cause of death if it died; and (4), survival and reproduction data as well as the occurrence of any problematic behaviour if the lynx was released in the wild. Cause of death was identified through necropsy effectuated by trained veterinarians. If no veterinarian was involved or the carcass was not fresh enough, we assigned the category “unknown” cause of death. The questionnaire was sent to 1–5 lynx experts per European country with confirmed lynx presence [13] and covered a period from 1970 to 2022. A lynx was defined as an orphan if it was an animal in its first year of life separated from its mother because (1) its mother died, (2) it failed to follow the mother, or (3) it was captured by humans because of true or assumed absence of the mother. Orphans were categorized into two age groups depending on the time of capture: small juveniles from June to August, i.e. still mostly depending on milk, and large juveniles from September to April. In contrast to Borel et al. [23], who considered both dead and living orphans, we took into account only orphans that were alive when first becoming conspicuous.

Two different survival rates were estimated: the first one concerned survival in captivity, measured over one year, or until released back into the wild. The second survival rate concerned post-release survival, measured over one year after release. The fate of the released lynx was monitored either by VHF or GPS collars or, if not collared or the collar stopped working, through camera trapping, as lynx are individually identifiable by their distinct coat pattern [27], or the retrieval of the carcass. Causes of mortalities were classified into confirmed illegal killing (lynx found with gunshot wounds, radio-transmitter retrieved in strange places without lynx body nearby), probable illegal killing (lynx disappeared despite equipped with new transmitter and no signs of technical problems following Andrén et al. [28]), natural (disease, starvation, avalanche, violent interaction with other lynx), traffic, unknown trauma (lynx found with broken bones) and unknown cause. The proportion of cases of probable illegal killings might be overestimated as radio-collars may fail for other unknown reasons to send data [29]. Survivor curves were plotted by the Kaplan-Meier method [30] and visualized using the ‘survival’ and ‘survminer’ packages of R [31,32].

Cox proportional hazard models were used to investigate the association of survival time and the following predictor variables: age at capture, having received hunting training during rehabilitation or not, age at release, release weight, time in enclosure, release time, type of release and release location [32]. Age was measured in months, assuming that kittens were born at the end of May [18], and release mass was recorded. Release time was classified into three categories: spring release between January and May, representing the time of separation from the mother in nature [22], summer release between June and August, and autumn release from September to December. Two types of releases were compared: a ‘soft’ release involved a period of confinement of individuals at the release site with food and water supplementation until the lynx became acclimatized to the new environment. By contrast, a ‘hard’ release involved immediate animal release into the wild upon translocation to the release site [33,34]. Three types of release locations were considered: core population, edge of lynx distribution and no lynx presence. For this purpose, we used the European Environmental Agency 10 by 10 km grid and checked for reported lynx presence at the release site and occurrence in the surrounding eight grid cells. Cells were considered occupied if lynx were reported to be present in at least three years out of five, i.e. permanently occupied according to the last European-wide assessment before release [16] or if annual distribution maps were available, if lynx presence was confirmed in the specific cell [35]. (1) Lynx were released into the core area of a lynx population if the release cell as well as six or more of the surrounding cells were already occupied before the release. (2) Lynx were released into an uncolonized area if the release cell was not occupied before the release and at the most two of the surrounding eight cells were occupied. (3) All other scenarios meant that the lynx were released at the edge of an area with lynx occurrence.

Because not all predictors were available for all individual orphans, we used eight different models fitted to each of the predictors. All models were corrected for observation bias, differentiating between observations made through radio-telemetry versus camera trapping, genetic sampling or carcass recovery by adding the observation method as a second predictor in the model. Using eight different models for each predictor of interest enabled us to use the largest sample possible. We also fitted a multivariate model using all predictors at the same time. For all models, we give the ‘Akaike’s Information Criteria’ [36] for small sample sizes (AICc) calculated based on the model fitted to the same data set (n = 55).

Dispersal distances were calculated for lynx which were monitored for at least 6 months after release, with dispersal distance defined as the Euclidian distance between the release site and the last recorded location. After natural log transformation, we assessed differences in dispersal distances between sexes and release location by means of a linear regression using the R software [37]. Reproduction of rehabilitated orphans was ascertained if a female was observed followed by kittens and for males based on genetic proof (methods described in Mueller et al. [38]). Finally, we also documented abnormal behavioural traits of orphaned lynx, such as habituation to humans (i.e., orphans becoming used to human presence, so that they no longer show signs of stress in the presence of humans [39], or other conspicuous behaviour.

### Ethics statement

In Croatia handling of lynx orphans was approved by the Ministry of economy and sustainable development and was carried out in accordance with Ordinance on sanctuaries for wild animals (Gazette No 140/20).

In the Czech Republic, lynx orphans have been treated by animal rehabilitation centers, permitted by the Czech Ministry of Environment, according to § 5 paragraph 9, Law 114/1992 Coll., on Nature and Landscape Protection.

In France, the wildlife rescue center "Centre Athenas" is authorized to hold and rehabilitate European birds, mammals, reptiles and amphibians. Prefectorial decree N° DDAF N° IST 598 modified with decree N° 39 20140117CSPP and decree N° 3920190107CSPP. The center is also authorized to capture injured and orphaned lynx under ministerial decrees 2006/435/AUT, 2008/133/EXP, 2009/047/DEROG, DEROG 10/06/2011, 12/847DEROG (3 years), DEVL1714207A, TREL2218563A.

In Germany, permission to release up to 20 lynx in Rhineland-Palatinate was provided by the Ministry for the Environment, Agriculture, Food, Viticulture and Forestry (105–62 232/2013-3#4), and Lower Saxony and Bavaria issued permissions for capturing and handling lynx (33.11.42502-04-082/07, 33.14-42502-04-10/0201, 33.19-42502-04-14/1571, 33.19-42502-04-19/3229).

The orphaned lynx in Poland were rescued and rehabilitated in accordance with the Nature Conservation Act.

In Romania, the collection of orphan lynx from the wild is considered accidental capture, falling under the provisions of the Government Decision No. 323/2010 on the establishment of the monitoring system for accidental captures and killings of all species of birds, as well as of the strictly protected species listed in Annexes No. 4A and 4B of the Government Emergency Ordinance No. 57/2007 on the regime of protected natural areas, conservation of natural habitats, flora and fauna, and does not require any special capture or handling permits. The Wildlife Rehabilitation Centre–ACDB is authorized in treating and rehabilitating wild animals based on the Environmental authorization No. 3 /10.04.2009 (renewed with the Environmental authorization No. 77/30.05.2019) issued by The National Environmental Protection Agency.

In Slovakia, rehabilitation and breeding stations belong to rescue facilities established by the state. These are devices that must meet several conditions established in accordance with the *Act no*. *543/2002 Coll*. *on nature and landscape protection* of the Slovak Ministry of the Environment and relevant EU International Conventions as amended. Nature protection organizations, i.e. j. *The State Nature Protection of the Slovak Republic and the National Parks Administration*, *in accordance with § 65a and § 65b of the Nature Protection Act*, perform the function of a breeding and rehabilitation station, receive notifications about found disabled, dead or accidentally caught, injured or killed protected animals, determine how to deal with them and lead records about them. At the same time, they cover expenses related to the care of handicapped protected animals, if such care is provided by a breeding or rehabilitation station established outside of nature protection organizations. Veterinary care for orphans was provided in accordance with Veterinary care Amendment to the Act on Veterinary Care 272/2021.

In Switzerland, the federal government defines the framework of action for the lynx as a protected species (Art 7 Federal Act on hunting and the protection of wild mammals and birds). The handling of orphan lynx is the responsibility of the cantons (enforcement of legislation). Some cantons provided permits to the research team to assist in the handling of lynx (BE: 66/97, 8/00, 109/10, and 111/13 BE3/17+, VD: 1047.1), The rearing of young lynx for rehabilitation is commissioned by the cantons to competent, authorized stations.

In Ukraine, orphan handling was carried out in strict accordance with the «Regulations on zoological parks of national importance» (order of the Ministry of environmental protection and nuclear safety of Ukraine and the Ministry of culture and arts of Ukraine № 21/46), «Procedure for keeping and breeding wild animals in captivity or in semi-free conditions» (order of Ministry of environmental protection of Ukraine № 429) and laws of Ukraine: «About the protection of animals from cruel treatment» (3447-iv), «About the animal world» (2894-iii), «About the Red book of Ukraine» (3055-iii), «About the nature reserve fund of Ukraine» (2456-xii). The study did not require obtaining special permits or approval by protocols of the ethical commission.

All surgery was performed under Medetomidine, Acepromazine, Midazolam, Morphine and Ketamine anaesthesia, and all efforts were made to minimize suffering and stress. Animals were euthanized if they were found to have incurable life-threatening diseases or injuries. For this purpose, the animal lying under anesthesia was administered Pentobarbital by the veterinarian. In Croatia, France, Poland and Slovakia, orphan lynx were fed with live prey under regular protocols of the rehabilitation centres.

## Results

### Orphaned lynx

**Fig 2:**
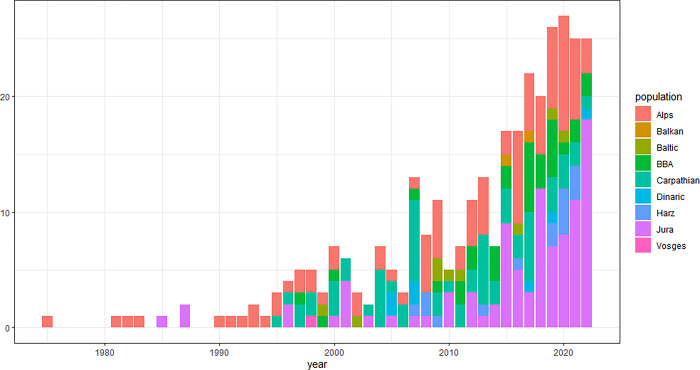
Development of the number of orphaned lynx reported in nine European populations per year.

Data on 320 orphaned lynx recorded from 1975 to 2022 were provided from 13 countries and nine populations (S1 Table). Most reports originated from Switzerland (n = 122), France (n = 70) and Slovakia (n = 50). The number of reported orphans in Europe varied from 0 to 27 per year, with an annual mean of 8 individuals and an increasing trend (Fig 2). Capture circumstances were reported for 192 lynx. Most were considered orphaned because they were found near buildings and/or were in a bad physical condition: 59 (31%) were captured inside villages, 38 (20%) near houses, including seven eating pet food, 15 (8%) were found inside an anthropogenic structure (barn, chicken pen, garage, washhouse) and twelve (6%) were captured in the forest. One kitten was rescued from drowning and another one was found alone inside a train tunnel. Forty-three cases (22%) of predation on domestic animals were reported shortly before capture (22 chicken or guineafowl, eight domestic cats, four rabbits, three goats, three lambs, two ducks and a dog). Ten kittens (5%) from eight litters were considered orphans due to the true or assumed absence of the mother and removed directly from the den and a kitten was separated from its mother due to a forest fire. Nine (5%) suffered injuries due to a trauma. Finally, three kittens were captured together with their mother who was injured in a traffic accident. After her recovery (17 days in captivity) the whole family group was released together.

**Fig 3:**
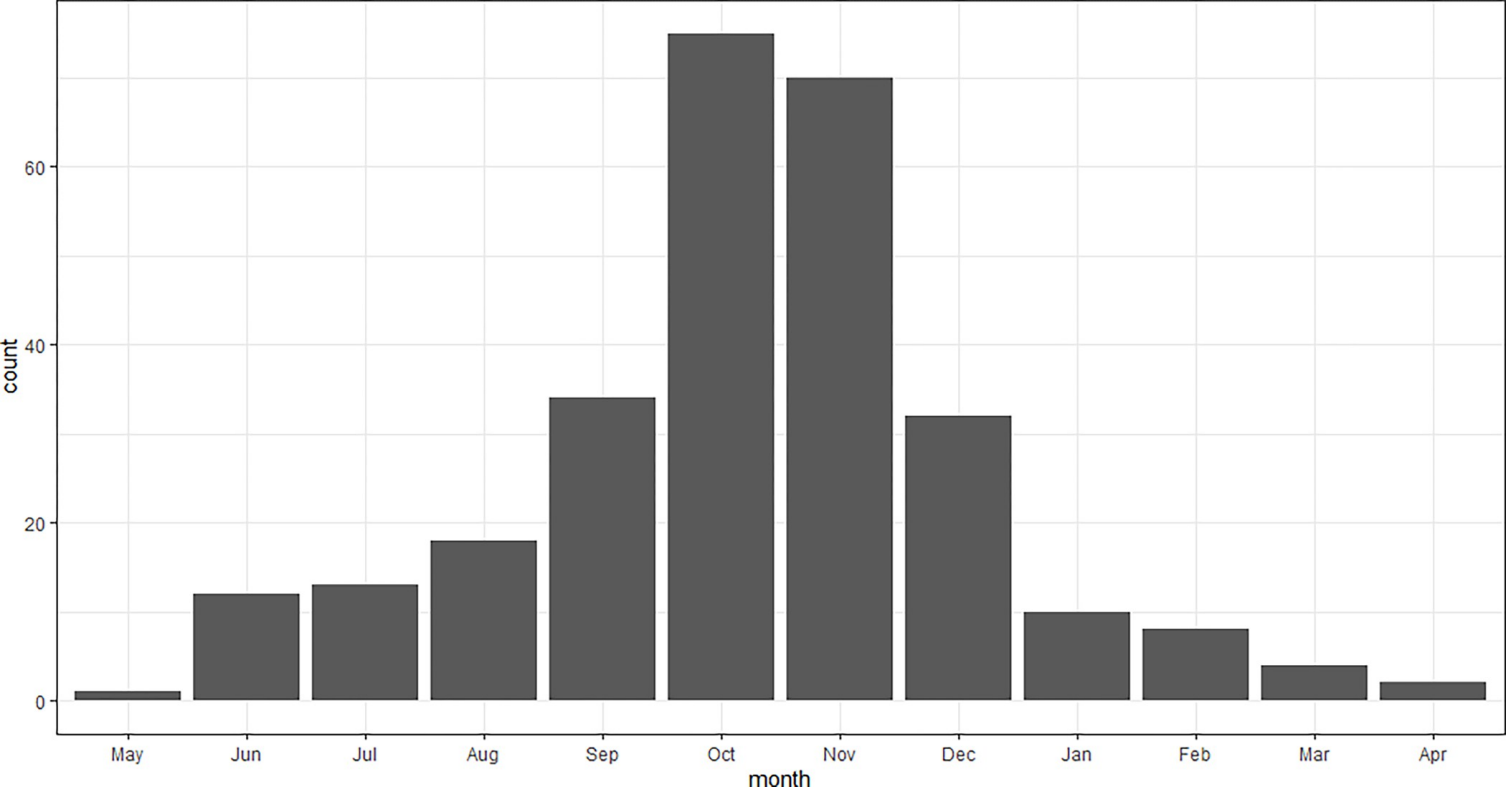
Number of orphaned lynx discovered per month.

Orphan age at capture ranged from few days to 11 months, most of them becoming conspicuous in October and November at the age of 5–6 months (Fig 3). Information on health status was available for 245 orphans: 172 (70%) were emaciated, 26 (11%) experienced trauma (13 had bone fractures, three had bite wounds, one suffered from burns, the rest had unspecified injuries), 13 (5%) had a disease and 34 (14%) were in good health. The fate of the mother was rarely known. Only 20 individuals (6%) from 12 litters were known to have lost their mother: seven mothers died in traffic accidents, three were illegally killed, one was probably illegally killed and the cause of death of one female was unknown. Three kittens (1%) were known to be abandoned, since it was confirmed by radiotelemetry that their mothers were alive.

### Orphan management

Orphan management differed depending on the situation: non-authorized persons immediately killed 17 orphans because they attacked domestic animals or were suspected to be infected by rabies (which was not confirmed by subsequent analyses), and 18 died before they were checked by a veterinarian. In Switzerland, 37 orphans were killed by the authorities (game wardens) due to a lack of a rehabilitation centre, a lack of political agreement to release after care in captivity and/or a lack of space in zoos [23]. Sixteen individuals were in very bad condition and euthanized by mandated veterinarians. For 39 orphans the decision was to do nothing or they disappeared before capture: nine of them died and three survived for at least one year. The fate of the other 27 was unknown. From 2005 to 2022, 19 orphans were fed in situ, of which six (32%) survived until 1^st^ March or longer (Table 1). Due to suboptimal locations (traffic, settlements close by), four orphans were captured and immediately released in more remote places three to six kilometres away from the capture site, where in-situ feeding was initiated. All other orphans were fed close to the place where they were found. For in-situ feeding, carcasses of roe deer, red deer or wild boar were used. The fate of most orphans who were fed in the wild remained unknown (Table 1).

**Table 1:** Implementation of in-situ feeding of lynx orphans in Europe. Time fed indicates the time in days between the position of the first carcass and the position of the last carcass. Monitoring was done by means of camera traps except ID1 which was captured and equipped with VHF collar.

ID	country	Date first observation	Date last observation	Feeding on carcass	Time fed (days)	Survived until 1. March
1	Croatia	25/10/2005	05/07/2006	yes	45	yes
2	Croatia	25/10/2005	27/10/2005	yes	45	?
3	Switzerland	27/07/2010	28/07/2010	yes	1	?
4	Switzerland	18/11/2015	25/11/2015	no		no (died in traffic accident)
5	Switzerland	08/10/2016	29/01/2019	yes	123	yes
6	Switzerland	06/12/2016	06/12/2016	no		?
7	Switzerland	15/02/2017	22/02/2017	yes	7	?
8	Switzerland	15/02/2017	24/02/2017	yes	9	?
9	Germany	14/10/2018	07/05/2019	yes	91	yes
10	Germany	14/10/2018	06/04/2019	yes	91	yes
11	Switzerland	14/12/2018	18/12/2018	yes	4	?
12	Switzerland	06/10/2020	09/11/2020	yes	34	?
13	Switzerland	06/10/2020	09/11/2020	yes	34	?
14	Switzerland	09/11/2020	09/11/2020	no		?
15	Switzerland	21/11/2020	04/03/2021	yes	103	yes
16	Switzerland	17/01/2021	06/03/2021	yes	48	yes
17	Switzerland	07/10/2021	09/10/2021	yes	2	?
18	Switzerland	18/10/2021	25/10/2021	yes	7	?
19	Switzerland	04/10/2022	12/10/2022	no		no (died emaciated)

**Fig 4:**
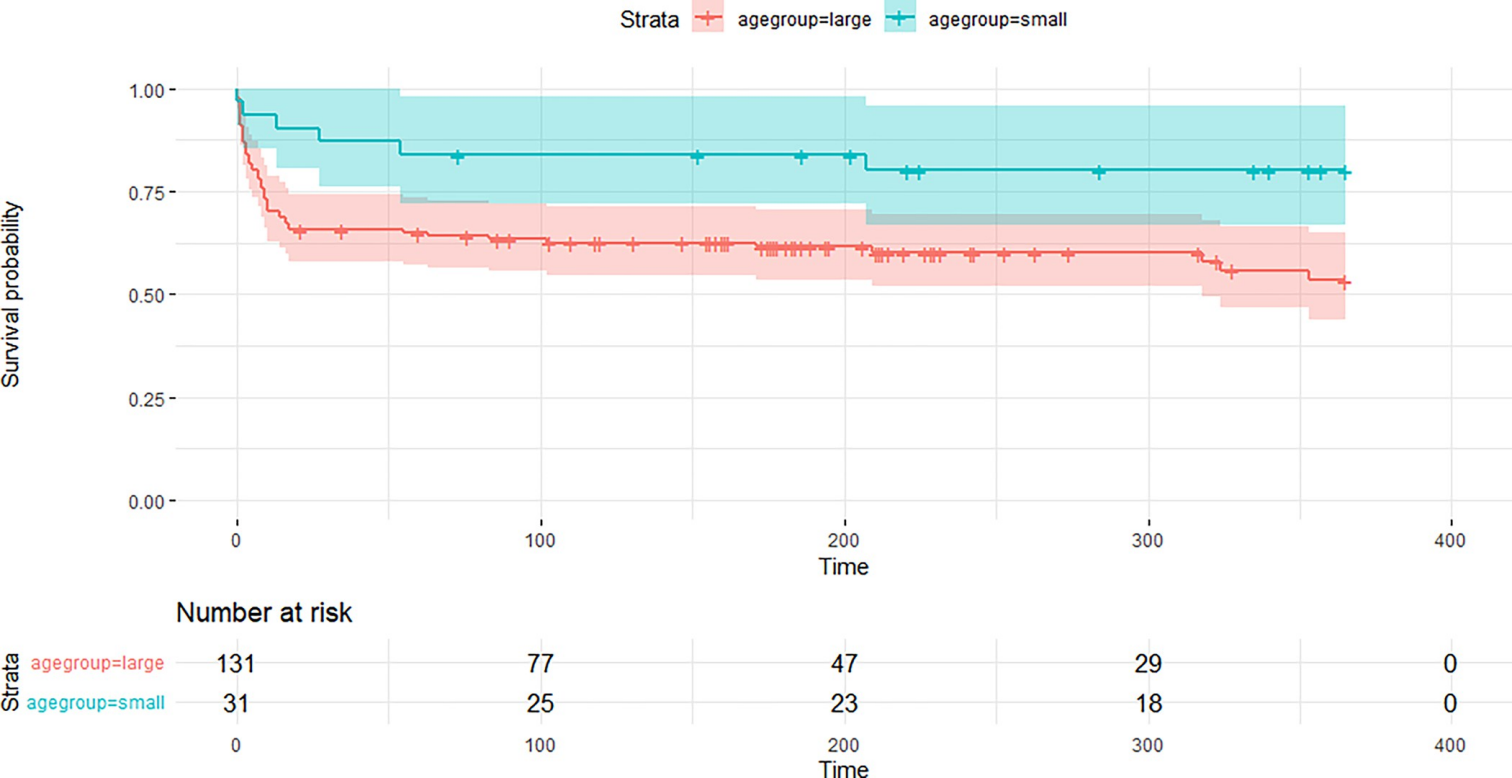
Kaplan-Meier survival curves of juveniles captured between June and August (small juveniles) and transferred to an enclosure (blue line) versus survival of juveniles captured between September and April (large juveniles) and transferred to an enclosure (red line).

The majority of orphaned lynx (174/320, 55%) were taken to rehabilitation centres or other enclosures, where 31/174 (18%) died within one week. The survival rate of one year after the capture was 0.60 with a total of 62 deaths. Most mortalities in care occurred as a direct result of the initial reason for admission, as 55/62 (89%) died due to previous health impairments including emaciation. Suboptimal enclosures or care were the cause of death of 4/62 individuals (6%). In captivity, small juveniles, captured between June and August, had a higher survival rate than orphans captured in September or later (Fig 4).

Thirty-one (18%) of the 174 orphans that were taken to rehabilitation centres were transferred to zoos for captive breeding programs, 75 individuals (43%) were planned to be released, including the three orphans that were captured together with their mother after a traffic accident. Four (2%) escaped from their enclosure: Two males escaped in October at the age of five months and were never seen again. A female escaped 21 days after being captured at the age of seven months: she survived for at least ten years and had several litters. Another female who spent two years in different enclosures survived for at least three years after her escape (Borel et al. 2022). However, she was often seen and photographed by people after her escape, likely a sign of habituation, and disappeared at the age of five years.

**Fig 5:**
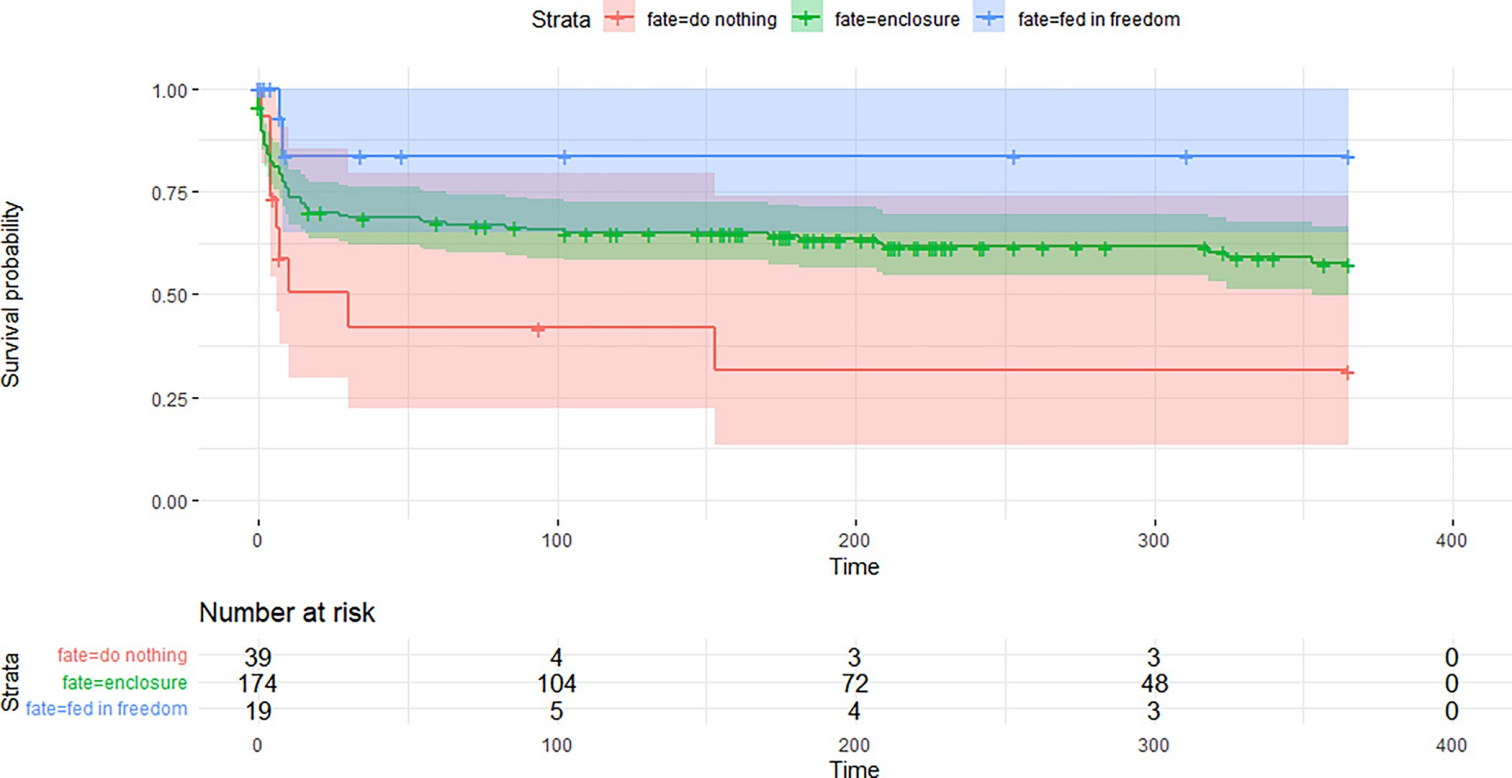
Comparison of survival rates of orphaned lynx based on different management options.

When comparing the survival rate of the three management options “no action taken”, “in-situ feeding” and “taking the orphan to an enclosure”, in-situ feeding ranked best (Fig 5). Unfortunately sample sizes for the two groups “no action taken” and “in-situ feeding” were very small and the fate of these orphans was rarely known. Only two orphans were known to have died in the group “in-situ feeding”, this being probably the reason for the high ranking of this management option.

### Release of orphans

From 1993 to 2023 in eight countries it was planned to release a total of 70 orphans (S2 Table). Based on the health check before release, it was decided not to release two of these lynx due to health impairments, one lynx died of a capture-related injury and another died during transport to the release site. The median time spent in rehabilitation centres by the released lynx was 209 days (range 76 days–four years). Median age and mass at release were 13 months (range eight months—four years) and 15 kg (range nine– 19 kg). Out of the 66 rehabilitated lynx, 33 were provided with live rabbits, rats, pigeons or guinea pigs prior to release as pre-release hunting training (in Croatia, France, Poland and Slovakia). Based on 60 released orphans monitored by radiotelemetry, the median time from release to first detected kill (for which the consumption lasted longer than 24 hours) was eight days (range two– 45 days). One orphan was recaptured 36 days after release because considered unable to hunt. Another one was recaptured after 71 days because it had killed six sheep. Hard release was used more often than soft release (54 versus twelve). Seven lynx were released in an unoccupied area, 14 at the distribution edge and 44 in a core area of a lynx population. Seven rehabilitated lynx were part of a reintroduction programme in the Palatinate Forest, Germany, where from 2016 to 2020 a total of 20 lynx were released [40]. Another three lynx were released at the edge of the population to accelerate population expansion. Apart from these ten rehabilitated lynx, who were translocated for reintroduction or reinforcement projects, release sites were chosen based on proximity to place of capture. Distance from roads and settlements as well as the agreement of the landowner were additional criteria that were considered. No measures were taken to improve public acceptance prior to release, with the exception of reintroduction and reinforcement projects, where collaboration with interest groups was established prior to release.

**Fig 6:**
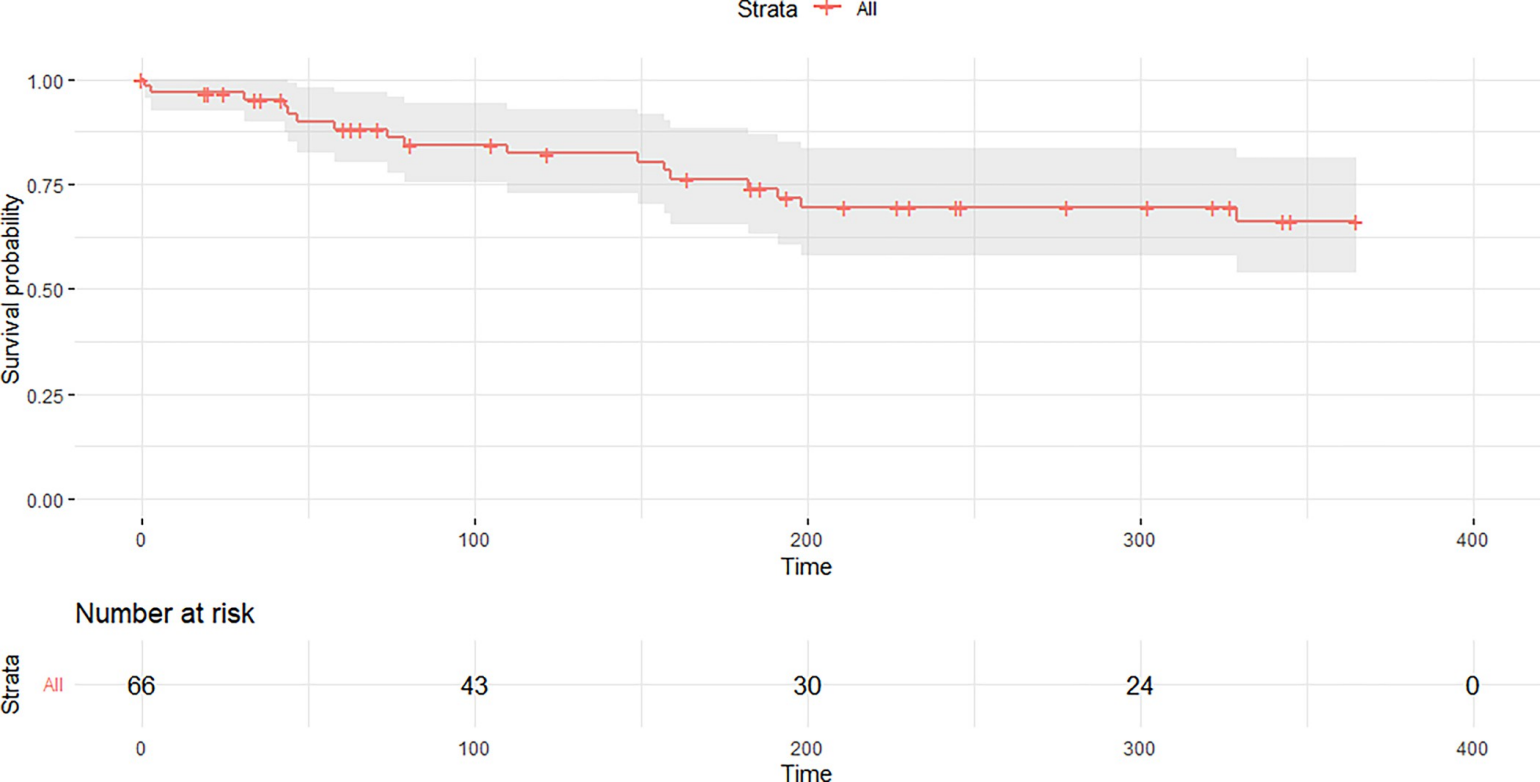
Kaplan-Meier survival curve of rehabilitated lynx in Europe over one year.

**Fig 7:**
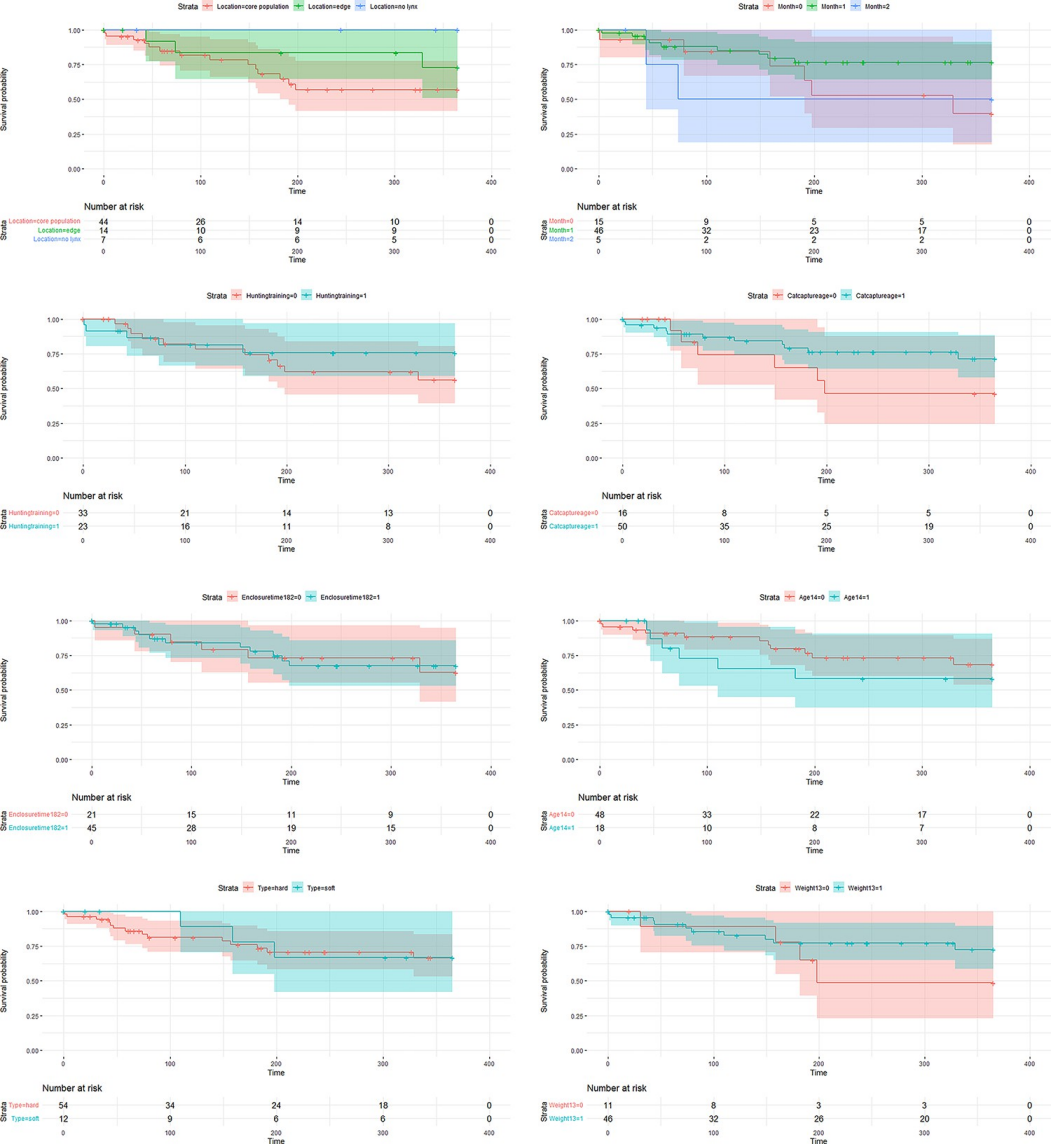
Comparison of survival rates of rehabilitated lynx based on different predictors. **A**) Release location based on lynx presence (see methods). **B**) Release month (0 = January to May, 1 = June to August, 2 = September to December). **C**) Having received hunting training (0 = no, 1 = yes). **D**) Age at capture (0 = captured between May and September, 1 = captured between October and April). **E**) Time in enclosure (0 = shorter than 182 days, 1 = 182 days or longer). **F**) Age at release (0 = younger than 15 months, 1 = 15 months or older). **G**) Soft versus hard release. **H**) Release weight (0 = lower than 13 kg, 1 = higher than 13 kg).

One year after release 17 orphans were known to be dead (proportion that survived = 0.66, Fig 6). Lynx released into areas with no other lynx present had on average six times (95% CI: 0.7–51.1) higher survival odds than lynx released in the core of the population distribution. Orphans released at the edge of lynx distribution still were 1.7 times (95% CI: 0.6–5.3) as likely to survive than lynx released in the core of the population distribution (Table 2, Fig 7). Lynx released during summer had almost twice as high odds to survive as lynx released during spring (odds ratio = 1.88, CI: 0.7–5.2) while lynx released in autumn have lower chance of survival than lynx released in spring (odds ratio = 0.74, CI: 0.1–4.1). Having received hunting training with live prey had an odds ratio of 1.98 (CI: 0.8–5.1). With each month the orphan was older at the time of capture, survival after release improved by 11% (odds ratio = 1.11, CI: 0.9–1.4). Survival increased by 11% (odds ratio = 1.11, CI: 0.9–1.5) with each kilogram the lynx was heavier when released. Soft release had an odds ratio of 0.81 (CI: 0.3–2.5) compared to hard release. Time in an enclosure and age at release had no effect (Table 2, Fig 7).

**Table 2:** Effects of parameters on survival of released orphaned lynx based on the eight single factor cox proportional hazard models and a multiple regression.

	Univariate models							Multiple regression		
Parameter	exp(-coef)	lower .95	upper .95	conc.	se (conc.)	AICc	Sample size	exp(-coef)	Lower .95	Upper .95
Location edge	1.71	0.55	5.28	0.68	0.05	112.3212	64	2.01	0.53	7.68
Location no lynx	5.92	0.69	51.05					3.42	0.20	57.09
Month of release 1	1.88	0.69	5.15	0.66	0.06	113.1391	65	1.76	0.37	8.32
Month of release 2	0.74	0.14	4.05					0.22	0.01	3.56
Hunting training	1.98	0.77	5.09	0.65	0.05	112.0662	64	1.52	0.38	6.01
Age at capture (month)	1.11	0.87	1.43	0.61	0.05	112.5159	65	0.72	0.27	1.91
Age at release (month)	1.00	0.92	1.08	0.62	0.06	112.6212	65	1.21	0.45	3.26
Weight at release (kg)	1.11	0.85	1.45	0.66	0.06	112.4787	56	1.11	0.78	1.57
Soft release (versus hard)	0.81	0.27	2.47	0.61	0.05	112.8368	65	1.06	0.24	4.82
Enclosuretime (week)	1.00	1.00	1.00	0.61	0.06	112.0904	65	1.00	0.97	1.02

For the categorical variable “location”, lynx released into the core area of the population is the reference. For “month of release”, lynx released from January to May were the reference (month of release 1 = June to August, month of release 2 = September to December). Concordance (conc.) is a measure of goodness of fit. If the model makes random guesses, we’d expect concordance to be 0.5. Sample size of the multiple regression was 55 and AICc 126.87. For making AICc values among univariate models comparable, we refitted each model to the same sample of complete cases (n = 55) to get the AICc values.

Of 17 rehabilitated orphans that died in the first year after release, the cause of mortality could be documented in 13 cases (illegal killing, n = 3; probable illegal killing, n = 3; unknown trauma, n = 2; disease, n = 2; avalanche, traffic and intraspecific killing, one each). Two lynx were released despite being suspected to be habituated and showed decreased escape behaviour in the presence of humans. One of them was often observed by the public and disappeared after 202 days. The other disappeared soon after release. Both were considered probably illegally killed.

**Fig 8:**
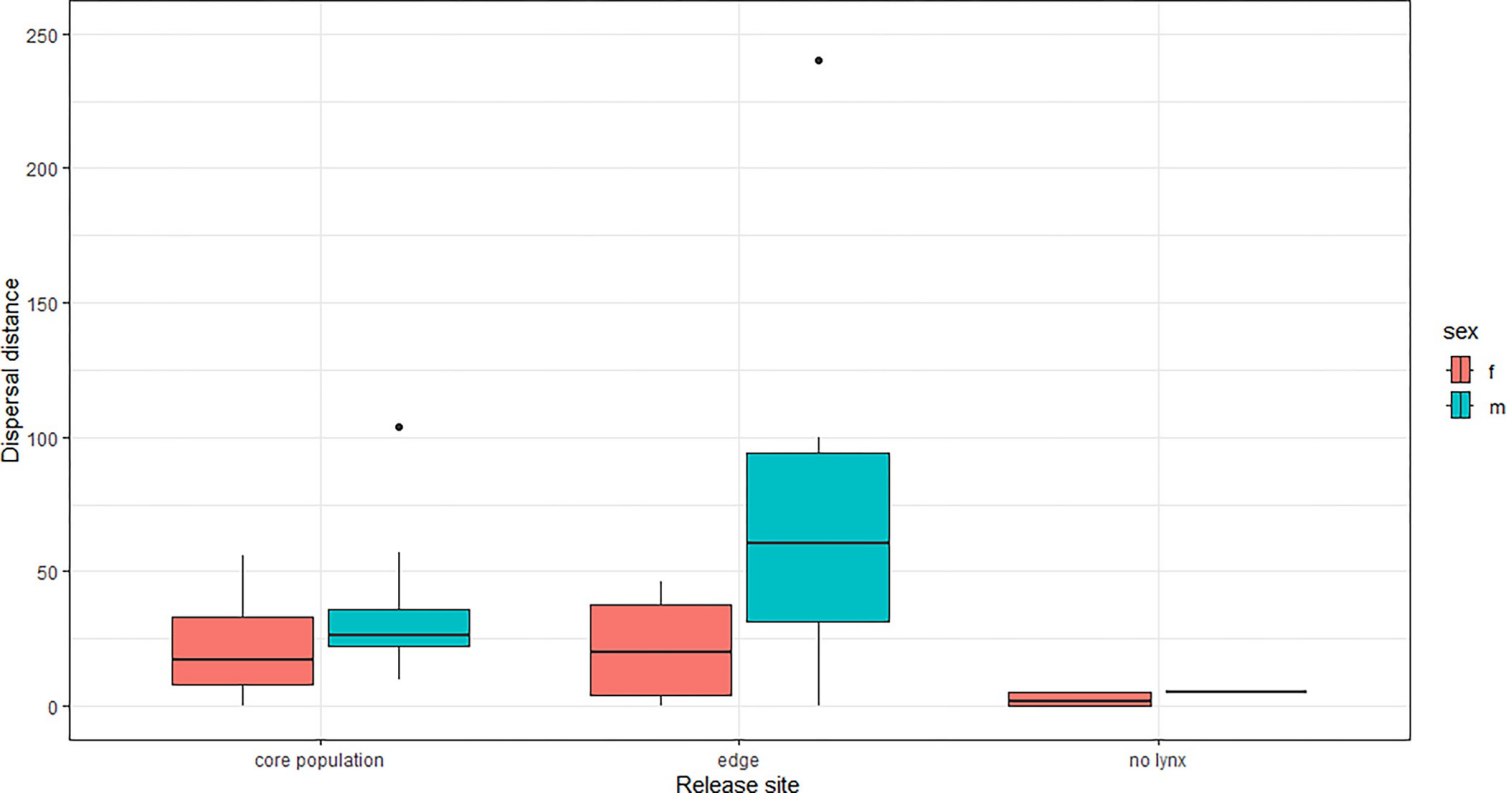
Dispersal distances of rehabilitated lynx depending on sex and release site occupancy.

The median dispersal distance of rehabilitated lynx was 23 km from the release site, with a maximum of 240 km. Males had 3 (range 1.1–7.5) times longer dispersal distances than females and lynx released into unoccupied areas had dispersal distances 0.2 (range 0.1–0.7) times shorter than lynx released into the core area (Table 3). All lynx who were released into unoccupied areas settled within 5 km of the release site (Fig 8). Of the 66 rehabilitated lynx, ten (8 females and 2 males) have reproduced at least once.

**Table 3:** Effects of sex and release location on dispersal distances.

Coefficients:	Estimate	Std. Error
(Intercept)	2.365	0.390
Sex m	1.072	0.473
Location edge	0.041	0.521
Location no lynx	-1.624	0.640

## Discussion

The fate of 320 orphaned lynx from nine different populations analysed in this study provided a unique opportunity to evaluate the feasibility of assisting lynx orphans until independence and re-integrating them into the wild lynx population. The trend of orphaned lynx occurrence in Europe is clearly increasing, in agreement with the positive population trends of some European lynx populations, improvements in monitoring and better communication tools (mobile phones, social networks). This increasing trend was most pronounced in the Jura Mountains (Fig 2), where lynx have recolonized almost all the suitable habitat available [41]. It is likely that in a saturated population lynx mothers may have more difficulty to care for their young while also traffic accidents and illegal killings of female lynx may become more frequent.

Orphaned lynx were mostly identified as such due to the presumed absence of their mother and/or their physical status. They were often found in proximity to human settlements, which together with a high lynx density [42] partly explains the high number of orphans reported from Switzerland. The mother was known to be dead in only a case minority and abandonment by the mother was confirmed only in three cases. Orphans were mostly discovered in October and November (when 5–6 months old), i.e., when they were already mobile enough to follow their mother but still too young to care for themselves.

Animal welfare and conservation considerations as well as other factors such as politics, financial aspects and rehabilitation centre availability have resulted in five different types of orphan lynx management approaches, from no action taken and killing, up to care—either in-situ or in captivity, sometimes with subsequent release back to the wild.

### No intervention

The chance of survival for orphans that are in no way assisted depended on age and health status. Natural separation from the mother peaks in April, but separations with subsequent survival of the juvenile lynx are reported already from the end of January [22]. At least four lynx were reported to be orphaned in December and survived without assistance. An exceptional case was observed in north-eastern Switzerland, where a lynx was separated from its mother at the end of October and survived until 1 April the next year, when it died in a traffic accident. Based on our data, juveniles becoming orphaned from December onwards may potentially survive, while lynx who lose their mother in September or earlier and are left in nature without any intervention are doomed to die. We lacked data about the survival probability of lynx orphaned in October and November, but the peak of orphans appearing in human settlements during these months (Fig 3) suggested strongly impaired survival during this season.

### Feeding in-situ

In-situ feeding of orphaned lynx provides the possibility for non-invasive support of juvenile lynx until independence. It has the advantage of being closer to a natural situation, meaning that the animal is not exposed to capture and captivity stress but goes through an experience that mimics natural separation from its mother. In addition, it reduces the risk of pathogen transmission [23,43] and habituation. On the other hand, health problems already carried by the orphans cannot be detected if no capture and no veterinary diagnostics are carried out. Feasibility of in-situ feeding depends on many factors such as age and health status of the orphans (no veterinary care required), the availability of carcasses of apparently healthy natural prey, social acceptance, the suitability of the location (distance to traffic lines, human settlements, scavengers such as brown bear, wolf or wild boar) and needs to be balanced against capturing and taking the orphan into a controlled environment. Although the management of in-situ feeding is cheaper than bringing the orphan to a rehabilitation center, it requires the availability of personnel who can organize the carcasses, manage the feeding and monitor the animal’s behavior. Although in-situ feeding had a higher survival probability than caring for the orphan in an enclosure or doing nothing, it needs to be taken into account that in-situ feeding was only applied when orphans were considered apparently healthy; and in-situ fed orphans were never tracked by means of radio-telemetry with one exception only which reduces the probability of monitoring the fate of these orphans. To increase the knowledge about the outcome of in-situ feeding lynx could be captured to equip them with a radio-collar in January/February, i.e. before their dispersal. Besides getting more knowledge about their fate it would also allow a veterinary check-up. If this is not feasible at least camera trap monitoring should be intensified to monitor the lynx until reaching the age of independence. In-situ feeding is preferable to taking no action, especially in areas with low lynx density. However, it should be conducted as long as needed, i.e. until the orphan can survive independently.

### Euthanasia

In a survey done by Guy, Curnoe & Banks [2] about current mammal rehabilitation and release practices, all respondents indicated that euthanasia was an acceptable option for animals with serious medical issues. Animal welfare and quality of life were common considerations, as were suffering, severe injury and low release likelihood. Despite the broad acceptance of mercy killing, in our study, this has been practised mainly in Switzerland (n = 13), with one case in France, Germany and Slovakia, respectively. In all countries, a lack of clear responsibility attributions and the hope of survival despite health problems have resulted in the avoidance of wildlife managers deciding to euthanize a lynx orphan. Nevertheless, it should be emphasized that euthanasia is the most reasonable decision when the animal’s welfare is compromised, for example, due to severe injuries, and when the prognosis is poor [3,44]. The potential distress experienced by animals needs to be pragmatically weighed with the benefits of survival for the individual and the release population [45].

### Transfer to rehabilitation centre/enclosure

Despite being often in unfavourable body condition when found, the probability of surviving up to twelve months of age of orphans taken to rehabilitation centres was 0.61 in captive settings, which was higher than juvenile survival in nature of 0.45–0.54 and 0.28–0.46 recorded in the north-western Alps of Switzerland and in Scandinavia, respectively [28,46]. The compiled data indicate that, with proper care, lynx orphans can be saved even when found at a very young age. However, nearly a fourth of the orphans taken to a rehabilitation centre died within one week due to previous health impairments, which suggests a need for triage criteria [44]. Early euthanasia not only prevents unnecessary animal suffering, it also prevents unnecessary use of limited resources. However, animal welfare, the orphan’s “genetic value” (see below) and financial aspects need to be evaluated case by case if a lynx cannot be released back to the wild, balancing the options of euthanasia, zoo display and/or captive breeding.

Transfer to rehabilitation centres implies capture and should in all cases include a veterinary clinical examination and initial triage [23]. Also, it should be considered if appropriate rehabilitation facilities and programs are available. Considering that only in five European countries lynx rehabilitation centres were registered, more enclosures should be adapted to be able to provide professional care for lynx orphans. Until those centres are available, we advise to consider transporting orphans to one of those rather than trying rehabilitation in an improvised enclosure and with inexperienced staff. If this transfer implies crossing national borders, procedures for authorizations need to be clarified beforehand. European countries that do not have appropriate lynx rehabilitation facilities and countries with a small number of orphans are encouraged to establish cooperation with facilities in other countries.

Special care needs to be taken to prevent habituation to humans of orphans that are supposed to be released back into nature. In order to reduce habituation and association of food with people, food should be provided while the individuals are not directly present to avoid direct contact with the keepers [47]. We strongly discourage the release of habituated lynx, as this might negatively impact the public perception.

### Release of orphans

Rehabilitation of Eurasian lynx orphans is a feasible practice that has been performed in eight European countries. Release variables varied greatly, with weights at release ranging from 9 to 19 kg and age from 8 months to more than 4 years. Accordingly, the time spent in captivity ranged from 76 days to 4 years. Age at orphaning and release mass as well as time spent in captivity was believed to be an important factor for rehabilitation in American black bear (*Ursus americanus)* and red fox (*Vulpes vulpes)* [9,48]. In our sample, release location was the best predictor for lynx survival, with lynx released into unoccupied areas having on average six times higher survival and lynx released at the edge of the distribution still had almost twice as high survival than lynx released into the core of the lynx population distribution (Table 2, Fig 7). Timing of release was also important: lynx released during summer had a twice as high odds to survive as lynx released during spring. Survival also increased by 11% with each kg the lynx was heavier when released. The effects of age at capture, age at release and type of release remained unclear, while time spent in enclosures had no effect (Table 2).

Another factor with positive influence on the survival of the rehabilitated lynx was hunting training, with lynx having received hunting training having a higher survival probability. Some countries allow providing orphaned lynx with live prey in order to learn hunting techniques. Those orphans had a higher survival probability during the first year after release. However, all orphans but two were able to hunt upon release despite more than half did not get training on live prey, demonstrating that such training is not essential for Eurasian lynx. Starvation was never reported as a mortality cause of the rehabilitated orphans, although cause of mortality was not known in four cases.

Traffic accidents, illegal killing and probable illegal killing were reported as the main mortality causes in the first year after the release. Confirmed and probable illegal killing accounted for 42% of the known mortality. The release of orphans should be accompanied by an information campaign to improve and maintain public acceptance among all interest groups. Rehabilitation provides wildlife authorities and rehabilitators an opportunity to resolve orphan issues in a positive light, and to educate and engage the public in wildlife management [49]. Social media facilitate the sharing of heart-warming stories and promote support for wildlife [50]. However, the success of orphan rehabilitation will often be dictated by the preliminary work done in developing a defensible plan and garnering support from the appropriate agencies, interest groups and the public [12].

Dispersal distance of orphans (median 23 km) was slightly lower than natal dispersals of 26 km reported for lynx in the Alps and lower than 63 km for lynx in the Jura Mountains [22]. First-year survival of released orphans (0.66) was similar to the survival of lynx aged 1–2 years in natural conditions, i.e., lynx that separate from their mothers and search for a territory (0.57–0.81 in Scandinavia, 0.6 in the Alps and 0.53 in the Jura Mountains [28,46,51].

### Conservation value of orphaned lynx

Orphan management options were ranked based on their contribution to lynx conservation: 1) Mercy killing and taking no action have no and transfer to a zoo for display has low conservation value. 2) Release of rehabilitated orphans into the wild and orphans transferred to a captive breeding centre have a high conservation value. 3) Rehabilitated orphans which are translocated to be released at the edge of a population or even in a different population have the highest conservation value. Conservation implications of rehabilitation programmes include the management of genetic diversity in small, isolated populations as well as species reintroduction to previously occupied habitats [40,49]. In the future, captive care and release of orphaned lynx could also contribute to the genetic restoration of lynx populations (44). All European populations are currently at least partially monitored, including DNA-based methods. Especially the wild-sourced reintroduced populations are facing low genetic diversity and partly severe inbreeding, which could be detrimental to population viability [38,52,53]. The significantly lower observed heterozygosity in reintroduced compared to most of the autochthonous populations indicates that reintroduction bottlenecks, isolation and post-release management have long-term consequences on the genetic composition of populations [38]. Given their current status, reintroduced lynx populations need rigorous management, which incorporates genetic evaluation and if necessary action as a critical component to support population health and viability. Habitat connectivity between European lynx populations needs to be improved to achieve genetic exchange. Where habitat connectivity and the resulting linkage of populations by natural dispersal cannot be achieved quickly enough, other measures are required. In such cases, the Bonn Lynx Expert Group [15] advised restocking of Eurasian lynx populations, especially those with high levels of inbreeding. Orphaned lynx rehabilitation could be crucial for coordinated lynx management in Central Europe. Orphaned lynx which can be chosen based on their genetic identity could be released in other reintroduced populations, mimicking natural gene flow between otherwise isolated populations. Gustafson et al. [54] have found that even a single successful migrant enhanced the genetic diversity of an inbred puma (*Puma concolor*) population in the Santa Ana Mountains, California (USA). The most prominent example of genetic rehabilitation through introducing individuals from another population stems from the Florida panther (*Puma concolor coryi*), where the release of few individuals substantially increased genetic diversity [55]. Lastly, saving orphans from highly threatened autochthonous populations such as the Balkan one, can contribute towards the recovery of the subspecies and the genetic viability if the release site is carefully planned [56]. The use of orphan lynx versus wild-caught individuals for the reintroduction and reinforcement projects could mitigate the pressure on vulnerable wild populations that were used as a source until now. Our results showed that in the past three years, at least ten orphans would have been available annually as potential candidates for genetic management.

Orphaned lynx have already contributed to lynx conservation in Central Europe: 1) The Palatinate Forest lynx population was founded with the release of 20 lynx between 2016–2020 from Switzerland and Slovakia, out of which seven were captured as orphans [40]. 2) In north-eastern Switzerland, a rehabilitated female was released close to the capture site where no female lynx was formerly documented. This area is separated from the core population by the Rhine valley. Previously documented dispersals of lynx crossing the Rhine valley concerned only male lynx. The rehabilitated female was therefore of crucial importance for local reproduction and the eastward expansion of the north-western Alpine lynx population. 3) Another two female orphans were released in northeastern Bavaria to establish a new stepping-stone population north of the Bavarian-Bohemian-Austrian lynx population, where they successfully reproduced, contributing to the northward expansion of this population.

## Conclusions

One of the most important challenges in rehabilitation is the assessment of outcomes (2). Long-term monitoring of rehabilitated animals is crucial to evaluate survival and reproduction but is challenging; therefore, published studies are rare. Most of them cover only one to three months after the release [23]. Lack of funds, insufficient staff, and collar failure all limited post-release monitoring of lynx raised in captivity. However, assessment is vital to any release project [57] as it informs rehabilitators if their practices are effective, contributing to conservation and improving welfare. The conservation value of releasing orphaned lynx can only be assessed when their actual contribution to the release population is known, i.e., when reproduction can be documented. The data gathered in the framework of this study are promising but systematic telemetry of released orphans is necessary to better understand the usefulness of lynx orphan rehabilitation. Modern techniques such as GPS collars should help overcome challenges associated with landscape structure and manpower. Collars should be programmed to cover at least the first potential reproductive season.

Wildlife managers in Europe will continue having to deal with orphan lynx in the future. Therefore, research must continue to develop our understanding of factors improving lynx rehabilitation. Documenting each step of the rehabilitation, release, and post-release stages and analysing the resulting data to continually make improvements in the rehabilitation protocols is imperative. A proactive approach should be chosen, where dealing with orphan lynx is discussed with interest groups and subsequently, clear guidelines on how to manage them should be included in lynx management plans. When dealing with an orphan, managers need to make a decision based on its health, age, location, the population of origin and the availability of a suitable rehabilitation centre [23]. Based on the present state of knowledge, it is recommended to transfer lynx to a rehabilitation centre provided that the lynx is either rehabilitated, integrated into a captive breeding program, or euthanized in case of health problems. If no rehabilitation centre is available and/or there is a lack of personnel and funding, in-situ feeding is the second best option, capable of bridging a few months until the orphan can provide for itself. Whenever feasible, capturing and collaring orphans who are fed in-situ is recommended to obtain more data about the success of this method. We cannot ignore an orphan’s fate, it is important to learn what went right and wrong so that pitfalls and shortcomings in future orphan management can be avoided [58].

Rehabilitation programmes have conservation implications that extend beyond individual welfare benefits [49]. It is recommended to release orphans at the distribution edge or in the frame of reintroduction projects instead of a release in the core area of a population where it is not necessary from a demographic and genetic point of view. Indeed, besides translocating wild-sourced individuals or reintroducing captive-bred animals, the release of translocated orphans could be a valuable contribution to Eurasian lynx conservation in Central Europe. A coordinated approach that operates on the metapopulation level and considers conservation aspects should be applied in the future.

## Supporting information

S1 Table Data collected about orphans from nine populations in Europe from 1975 to 2022. (XLSX)

S2 Table Characteristics of rehabilitated orphaned lynx in Europe in the period 1994–2022. Individuals who are known to have reproduced are marked in bold. Definitions of “Type” and “Location” can be found in Methods.
(DOCX)
