# Supplementary material for: Rehabilitation and release of orphaned Eurasian lynx (Lynx lynx) in Europe: Implications for management and conservation
Source: PLoS One. 2024 Mar 7;19(3):e0297789. doi: 10.1371/journal.pone.0297789 (PMC10919842; doi:10.1371/journal.pone.0297789)
Supplement: S2 Table — Individuals who are known to have reproduced are marked in bold. Definitions of “Type” and “Location” can be found in Methods. (DOCX) [file pone.0297789.s002.docx]

**S2 Table. Characteristics of rehabilitated orphaned lynx in Europe in the period 1994-2022. Individuals who are known to have reproduced are marked in bold. Definitions of “Type” and “Location” can be found in Methods. Two orphans are still in an enclosure.**

| Nr | Date of release | Age at capture (months) | Age at release (months) | Weight (kg) | Time in enclosure (d) | Type | Location | Time to first kill (d) | Dispersal distance (km) | Duration of telemetry surveillance | Date of last observation | Time of survival (days) | Fate (cause of mortality/reason for recapture/euthanasia) |
| --- | --- | --- | --- | --- | --- | --- | --- | --- | --- | --- | --- | --- | --- |
| 1 | 09/04/1994 |  |  |  |  |  |  |  |  |  |  |  | not released/euthanasia (health) |
| 2 | 29/06/1999 | 6 | 13 |  | 220 | hard | no lynx |  | 2 | 343 | 06/06/2000 | 343 | unknown |
| 3 | 24/03/2003 | 3 | 10 |  |  | hard | core population |  |  | 191 | 01/10/2003 | 191 | dead (illegally killed) |
| 4^1^ | 21/04/2006 | 5 | 22 | 14 | 553 | hard | core population | 5 |  | 66 | 26/06/2006 | 66 | unknown |
| 5^1^ | 13/07/2006 | 2 | 14 | 18 | 353 | hard | unknown |  |  | 36 | 18/08/2006 | 36 | recaptured (unable to hunt) |
| 6^1,2^ | 28/03/2008 | 5 | 10 | 9 | 160 | soft | edge |  |  |  | 28/03/2008 | 0 | unknown |
| 7^1^ | 28/03/2008 | 2 | 10 | 9 | 225 | soft | edge |  |  | 20 | 17/04/2008 | 20 | unknown |
| 8^1^ | 14/05/2008 | 7 | 12 | 16 | 161 | hard | edge | 16 |  | 157 | 18/10/2008 | 157 | dead (probably illegally killed) |
| 9 | 13/04/2009 | 6 | 11 | 17.9 | 147 | soft | edge | 6 | 100 | 301 | 09/02/2010 | 302 | unknown |
| **10** | **13/04/2009** | 6 | **11** | **14.5** | **147** | **soft** | **edge** |  | **46** | **560** | **25/10/2010** | **560** | **unknown (R)** |
| 11^1^ | 10/06/2009 | 6 | 13 | 18 | 195 | hard | edge | 23 | 35 | 278 | 15/03/2010 | 278 | unknown |
| 12 | 05/06/2010 | 7 | 13 | 11 | 183 | hard | core population | 8 |  | 31 | 06/07/2010 | 31 | dead (probably illegally killed) |
| **13^1^** | **13/06/2011** | 6 | **13** | **16** | **211** | **hard** | **core population** | **20** | **56** | **366** | **15/08/2020** | **3351** | **alive (R)** |
| 14^1^ | 13/06/2011 | 7 | 13 | 17 | 181 | hard | edge | 15 |  | 43 | 26/07/2011 | 43 | dead (illegally killed) |
| **15** | **21/05/2012** | 5 | **12** | **13.7** | **206** | **hard** | **edge** | **8** | **5** | **319** | **17/10/2019** | **2705** | **alive (R)** |
| 16 | 13/05/2013 | 4 | 12 | 14.7 | 227 | hard | core population |  |  | 71 | 23/07/2013 | 71 | recaptured (killed livestock) |
| 17 | 21/05/2013 | 7 | 12 | 14.3 | 162 | hard | edge | 4 | 76 | 417 | 02/01/2015 | 591 | dead (traffic) |
| 18 | 27/05/2013 | 7 | 12 | 11.3 | 162 | hard | core population |  | 17 | 194 | 07/12/2013 | 194 | unknown |
| 19^1^ | 29/06/2013 | 6 | 13 |  | 229 | hard | core population | 17 | 18 | 186 | 01/01/2014 | 186 | unknown |
| 20 | 01/07/2013 | 3 | 25 |  | 699 | hard | core population |  |  | 42 | 12/08/2013 | 42 | unknown |
| **21** | **03/04/2014** | 7 | **11** | **16.2** | **120** | **soft** | **edge** | **42** | **35** | **500** | **28/09/2015** | **543** | **dead (unknown, R)** |
| 22^1,2^ | 18/07/2014 | 8 | 13 |  | 184 | hard | core population |  |  |  | 18/07/2014 | 0 | unknown |
| 23^1^ | 09/09/2014 |  |  |  |  |  |  |  |  |  |  |  | not released (died during transport) |
| 24^1^ | 31/05/2016 | 5 | 12 | 16 | 243 | hard | unknown |  | 36 | 246 | 01/02/2017 | 246 | unknown |
| 25^1^ | 01/06/2016 | 7 | 13 | 16.5 | 160 | hard | unknown |  |  | 122 | 01/10/2016 | 122 | unknown |
| 26^1^ | 13/07/2016 |  |  |  |  |  |  |  |  |  |  |  | not released (health) |
| 27^1^ | 13/07/2016 |  |  |  |  |  |  |  |  |  |  |  | not released (health) |
| **28^1^** | **30/07/2016** | 8 | **14** | **14.7** | **317** | **hard** | **no lynx** | **5** | **5** | **1017** | **13/05/2019** | **1017** | **dead (traffic, R)** |
| 29^1^ | 30/07/2016 | 6 | 49 | 16.8 | 1650 | hard | no lynx |  | 5 | 142 | 01/04/2017 | 245 | unknown |
| **30^1^** | **30/07/2016** | 4 | **37** | **15.25** | **982** | **hard** | **no lynx** |  | **5** | **363** | **01/06/2020** | **1402** | **unknown (R)** |
| **31** | **07/08/2016** | 7 | **15** | **11.5** | **232** | **hard** | **no lynx** | **7** | **0** | **2** | **01/12/2020** | **1577** | **alive (R)** |
| 32 | 02/06/2017 | 1 | 25 |  | 710 | hard | core population |  | 98 | 47 | 19/07/2017 | 47 | dead (unknown) |
| 33^3^ | 02/06/2017 | 1 | 25 |  | 715 | hard | core population |  | 43 | 58 | 30/07/2017 | 58 | dead (unknown) |
| 34 | 13/07/2017 | 5 | 14 | 12.8 | 263 | hard | core population | 16 | 13 | 182 | 11/01/2018 | 182 | dead (avalanche) |
| **35** | **13/07/2017** | 5 | **14** | **11.2** | **263** | **hard** | **core population** | **11** | **0** | **237** | **22/10/2018** | **466** | **unknown (R)** |
| 36^1^ | 15/12/2017 | 3 | 19 | 19 | 490 | hard | edge | 7 |  | 74 | 27/02/2018 | 74 | dead (traffic) |
| 37 | 20/12/2017 | 6 | 19 | 16 | 399 | hard | edge |  |  | 44 | 02/02/2018 | 44 | dead (trauma) |
| 38^3^ | 29/01/2018 | 2 | 8 | 12 | 202 | soft | core population | 6 | 57 | 198 | 15/08/2018 | 198 | dead (probably illegally killed) |
| 39 | 22/04/2018 | 5 | 11 | 14 | 176 | hard | edge | 8 | 0 | 250 | 17/03/2019 | 329 | dead (natural) |
| 40^1^ | 13/05/2018 | 5 | 12 | 12.5 | 211 | hard | core population | 3 |  | 90 | 24/10/2018 | 164 | unknown |
| **41^1^** | **11/09/2018** | 5 | **16** | **16.7** | **323** | **hard** | **core population** | **6** | **10** | **406** | **03/08/2020** | **692** | **unknown (R)** |
| 42^1^ | 12/09/2018 | 4 | 16 | 16.1 | 372 | hard | core population | 7 |  | 544 | 01/09/2020 | 720 | unknown |
| 43^1^ | 02/05/2019 | 6 | 12 | 17 | 158 | hard |  |  |  | 3 | 05/05/2019 | 3 | dead (disease) |
| 44^2^ | 08/05/2019 | 9 | 12 | 11.5 | 76 | soft | core population |  | 33 |  | 03/07/2020 | 422 | dead (traffic) |
| 45 | 11/06/2019 | 5 | 13 | 13.3 | 253 | hard | edge | 11 | 26.5 | 183 | 11/12/2019 | 183 | unknown |
| 46 | 29/02/2020 | 6 | 9 | 13 | 110 | soft | core population |  |  | 94 | 06/08/2020 | 159 | dead (unknown) |
| 47^1^ | 23/04/2020 | 5 | 11 | 16 | 189 | hard | core population | 10 |  | 105 | 06/08/2020 | 105 | unknown |
| 48^1^ | 29/04/2020 | 5 | 11 | 17 | 186 | hard |  |  |  | 1 | 30/04/2020 | 1 | dead (disease) |
| 49^1^ | 04/05/2020 | 6 | 12 | 16.1 | 173 | hard | edge |  | 240 | 558 | 13/11/2021 | 558 | alive |
| 50 | 19/05/2020 | 4 | 12 | 16 | 290 | hard | core population |  | 25 | 464 | 15/03/2022 | 665 | alive |
| 51^1^ | 19/05/2020 | 6 | 12 | 16 | 195 | hard | core population | 14 | 104 | 327 | 11/04/2021 | 327 | alive |
| 52^1^ | 20/05/2020 | 6 | 12 | 15.3 | 175 | hard | edge |  | 45 | 299 | 20/05/2021 | 365 | alive |
| 53^2^ | 09/06/2020 | 6 | 13 | 13.2 | 215 | hard | core population |  |  |  | 09/06/2020 | 0 | unknown |
| 54^2^ | 19/06/2020 | 2 | 13 | 15 | 340 | soft | no lynx |  | 0 |  | 20/04/2022 | 670 | alive |
| 55 | 23/03/2021 | 7 | 10 | 14 | 103 | hard | core population |  |  | 79 | 10/06/2021 | 79 | dead (trauma) |
| **56^1^** | **26/04/2021** | 5 | **11** | **15** | **178** | **hard** | **core population** | **7** | **0** | **537** | **15/10/2022** | **537** | **alive (R)** |
| 57 | 14/06/2021 | 5 | 13 | 17.5 | 335 | hard | core population | 45 | 8 | 345 | 25/05/2022 | 345 | alive |
| 58 | 14/06/2021 | 2 | 13 | 14.8 | 230 | hard | core population | 12 | 22 | 345 | 25/05/2022 | 345 | alive |
| 59 | 15/06/2021 | 6 | 13 | 14.6 | 213 | hard | unknown | 41 |  | 227 | 28/01/2022 | 227 | alive |
| 60 | 07/07/2021 | 10 | 14 | 15.2 | 129 | soft | core population |  | 23 | 570 | 29/05/2023 | 691 | alive |
| 61 | 22/07/2021 | 8 | 14 | 14.7 | 177 | soft | core population |  | 10 | 110 | 09/11/2021 | 110 | dead (unknown) |
| 62^1^ | 10/05/2022 | 4 | 12 | 16 | 242 | hard | core population | 3 |  | 149 | 06/10/2022 | 149 | dead (illegally killed) |
| 63^1^ | 12/05/2022 | 6 | 12 | 16 | 155 | hard | core population | 5 | 26 | 231 | 29/12/2022 | 231 | alive |
| 64^1^ | 08/06/2022 | 10 | 13 | 16 | 78 | hard | core population | 2 | 0 | 211 | 05/01/2023 | 211 | alive |
| 65 | 22/09/2022 | 4 | 16 | 17 | 344 | hard | core population | 8 |  | 25 | 17/10/2022 | 25 | unknown |
| 66 | 27/04/2023 | 5 | 11 | 13.5 | 156 | hard | core population |  |  | 81 | 17/07/2023 | 81 | alive |
| 67^1,2^ | 02/05/2023 | 5 | 12 | 15 | 194 | hard | core population |  |  |  | 21/05/2023 | 19 | alive |
| 68^1^ | 15/05/2023 | 5 | 12 |  | 201 | hard | core population |  |  | 63 | 17/07/2023 | 63 | alive |
| 69^1^ | 17/05/2023 | 7 | 12 |  | 164 | hard | core population |  |  | 61 | 17/07/2023 | 61 | alive |
| 70^1^ | 13/06/2023 | 5 | 12 | 14 | 212 | soft | no lynx | 15 |  | 34 | 17/07/2023 | 34 | alive |

^1^ has received hunting training

^2^ not radio-collared

^3^ habituated
